# An Improved Zero Potential Circuit for Readout of a Two-Dimensional Resistive Sensor Array

**DOI:** 10.3390/s16122070

**Published:** 2016-12-06

**Authors:** Jian-Feng Wu, Feng Wang, Qi Wang, Jian-Qing Li, Ai-Guo Song

**Affiliations:** School of Instrument Science and Engineering, Southeast University, Nanjing 210096, China; 220162828@seu.edu.cn (F.W.); 220162762@seu.edu.cn (Q.W.); ljq@seu.edu.cn (J.-Q.L.); a.g.song@seu.edu.cn (A.-G.S.)

**Keywords:** the 2-D resistive sensor array, zero potential circuit, improved zero potential circuit, measurement, crosstalk

## Abstract

With one operational amplifier (op-amp) in negative feedback, the traditional zero potential circuit could access one element in the two-dimensional (2-D) resistive sensor array with the shared row-column fashion but it suffered from the crosstalk problem for the non-scanned elements’ bypass currents, which were injected into array’s non-scanned electrodes from zero potential. Firstly, for suppressing the crosstalk problem, we designed a novel improved zero potential circuit with one more op-amp in negative feedback to sample the total bypass current and calculate the precision resistance of the element being tested (EBT) with it. The improved setting non-scanned-electrode zero potential circuit (S-NSE-ZPC) was given as an example for analyzing and verifying the performance of the improved zero potential circuit. Secondly, in the S-NSE-ZPC and the improved S-NSE-ZPC, the effects of different parameters of the resistive sensor arrays and their readout circuits on the EBT’s measurement accuracy were simulated with the NI Multisim 12. Thirdly, part features of the improved circuit were verified with the experiments of a prototype circuit. Followed, the results were discussed and the conclusions were given. The experiment results show that the improved circuit, though it requires one more op-amp, one more resistor and one more sampling channel, can access the EBT in the 2-D resistive sensor array more accurately.

## 1. Introduction

The two-dimensional (2-D) resistive sensor arrays were used in artificial electronic skin [1], tactile sensors [2,3], chemical sensors [4], imaging sensors [5], human-machine interaction input devices [6], structural health monitoring tools [7], etc. For accessing all elements in the *M* × *N* resistive sensor arrays with low complexity, many readout circuits, including the inserting diode circuit [8,9], the inserting transistor circuit [5,10], the passive integrator circuit [11,12], the voltage feedback circuit (VFC) [4,6,13,14,15,16], and the zero potential circuit (ZPC) [2,3,17,18,19,20,21], were proposed with *M* shared row wires and *N* shared column wires, in which one end of each element was connected with one shared row wire and the other end of the element was connected with one shared column wire. In these readout circuits, the VFC and the ZPC were applied more widely. In the shared row-column fashion, these circuits needed fewer wires but suffered from the crosstalk problem for the non-scanned elements’ bypass currents, which had more significant effect with the increase of the current values. The bypass currents would increase with the decrease of the resistances of the non-scanned elements and the increase of array size. The effect of the bypass current was also influenced by the multiplexer’s switch-on resistance (*R_switch_*). Based on the VFC, with extra resistors and extra sampling channels, the improved isolated drive feedback circuit (IIDFC) [15] and the IIDFC with compensation [16] were proposed for suppress the crosstalk caused by *R_switch_*s. The two-wire circuits, such as the two-wire VFC [22], the two-wire setting non-scanned-driving-electrode equipotential circuit (S-NSDE-EPC) [23], the multi-channel part two-wire ZPC [24], and the multi-channel full two-wire ZPC [25], were proposed for suppressing the crosstalk for long cables, and good performances were obtained. However, in these two-wire circuits, many extra components, including many wires and many operational amplifiers (op-amps), were necessary. The traditional ZPCs had simple structures but suffered from the crosstalk problem for the *R_switch_*, the resistances of the non-scanned elements, and array size. The ZPC with high accuracy and low complexity is still lacking.

In this paper, we hope to propose a novel improved zero potential circuit (IZPC) with high accuracy and a simple structure for suppressing the crosstalk problem. It uses two op-amps in negative feedback, two feedback resistors, and two sampling channels in the resistive sensor array. Following this introductory section, Section 2 introduces design and the principle analysis of the IZPC. Section 3 presents results of experiments. Section 4 presents a discussion of the results, which is followed by conclusions in Section 5.

## 2. Design and Principle Analysis of the Improved Zero Potential Circuit

**Figure 1:**
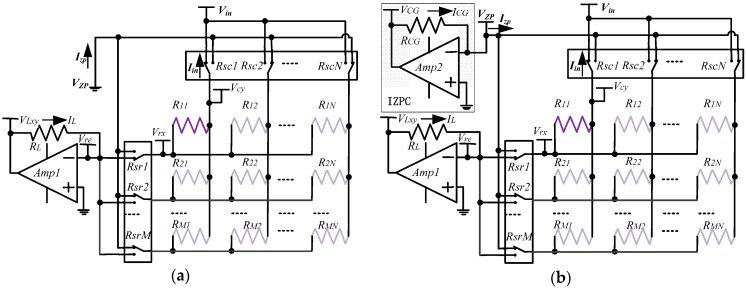
(**a**) Schematic of the basic S-NSE-ZPC; and (**b**) schematic of the improved S-NSE-ZPC.

Some ZPCs [17,20,21,23], each with one op-amp in negative feedback, were proposed to access all elements in the 2-D resistive sensor array, in which only one element could be selected and measured at the same time. Liu et al. [17] classified these basic ZPCs into the setting non-scanned-electrode zero potential circuit (S-NSE-ZPC), the setting non-scanned-sampling-electrode zero potential circuit (S-NSSE-ZPC), and the setting non-scanned-driving-electrode zero potential circuit (S-NSDE-ZPC). In this paper, we take the basic S-NSE-ZPC as shown in Figure 1a for example; similar analysis can also be applied in the S-NSSE-ZPC and the S-NSDE-ZPC.

In the basic circuit, as shown in Figure 1a, by switching on its 2:1 multiplexers (*R_sr_*_1_ and *R_sc_*_1_) and switching off all other 2:1 multiplexers (*R_sr_*_2_…*R_srM_*, and *R_sc_*_2_…*R_scN_*), the element being tested (EBT, *R*_11_) was selected and measured. Thus, the EBT’s row electrode and its column electrode were connected to the inverting input of the *Amp1* and the constant voltage source (*V_in_*) respectively, and all non-scanned electrodes were directly connected to a constant voltage (*V_zp_*) in the basic circuit. The *V_zp_* could be zero potential as shown in Figure 1a for the ZPCs [17,20,21] or another constant voltage for the equipotential circuits [23]. Through the scanning row 2:1 multiplexer (*R_sr_*_1_), the current (*I_L_*) on the feedback resistor (*R_L_*) was injected into the scanning row electrode of the array. Through all non-scanned 2:1 multiplexers, the total bypass current (*I_zp_*) from the *V_zp_* was injected into all non-scanned electrodes of the array. Through the scanning column 2:1 multiplexer (*R_sc_*_1_), the current (*I_in_*) from the array flowed to the *V_in_*. Therefore, in the basic circuit as shown in Figure 1a, based on Kirchhoff’s law of electric current, the current (*I_in_*) on the scanning column switch can be expressed as Equation (1) and the EBT’s resistance (*R_xy_*) can be calculated with Equation (2):

(1) $$I_{in}=I_{zp}+I_{L}$$

(2) $$R_{xy}=-\frac{V_{in}}{V_{Lxy}}R_{L}$$

where *V_Lxy_* is the output voltage of the *Amp1*.

With *Amp1*, the voltage (*V_rx_*) on the scanning row electrode can be expressed as Equation (3):

(3) $$V_{rx}=-\frac{V_{Lxy}}{R_{L}}R_{sr}$$

where *R_sr_* is the switch-on resistance of the row multiplexer.

In an ideal circuit, the switch-on resistances of the multiplexers were zero and the voltage on every electrode in the array was equal to the voltage on its gated terminal. Therefore, no bypass current existed on every non-scanned element (*R_non-scanned_*, resistive sensors in array except the EBT). However, in the real circuit as shown in Figure 1, the *R_switch_*s were not zero. Thus, there existed a voltage difference between the *V_re_* and the voltage (*V_rx_*) on the scanning row electrode and a voltage difference between the *V_in_* and the voltage (*V_cy_*) on the scanning column electrode. There also existed voltage differences between the *V_zp_* and the voltages on all of the non-scanned electrodes, which caused the *I_zp_* from the *V_zp_* injected into the array. The *I_zp_* changed with the *R_switch_*s’ variations and the non-scanned elements’ variations. Therefore, the crosstalk caused by the *R_switch_*s and the non-scanned elements affected the EBT’s measurement error. For suppressing the crosstalk in the improved circuit, we used another op-amp (*Amp2*) in negative feedback, as shown in Figure 1b, to sample the *I_CG_* from the *V_zp_* injected into all non-scanned electrodes of the array and keep the *V_zp_* equal to zero potential by a virtual short circuit. Therefore, based on Kirchhoff’s law of electric current, the *I_in_* can be expressed as Equation (4) in the improved circuit:

(4) $$I_{in}=I_{CG}+I_{L}=\frac{V_{CG}}{R_{CG}}+\frac{V_{Lxy}}{R_{L}}$$

where the *I_CG_* is the current on *R_CG_*.

Thus, the voltage (*V_cy_*) on the scanning column electrode can be expressed as Equation (5):

(5) $$V_{cy}=V_{in}+I_{in}R_{sc}=V_{in}+(\frac{V_{CG}}{R_{CG}}+\frac{V_{Lxy}}{R_{L}})R_{sc}$$

where *R_sc_* is the column multiplexer’s switch-on resistance.

Therefore, in the improved circuit, the accurate voltage drop (*V_cy_* − *V_rx_*) on the EBT can be obtained. Based on the basic principle of the traditional ZPC, with the multiplexers of small switch-on resistances, the difference between the voltage on each non-scanned column electrode and the *V_zp_* is small, and the currents on all row-adjacent elements are tiny. If we neglect the currents on all row-adjacent elements, the current on the EBT and the *I_L_* are equal. Thus, the measured resistance (*R_xy_*_1_) of the EBT in the improved circuit can be calculated with Equation (6) in the case of neglecting the small bypass currents on all row adjacent elements:

(6) $$R_{xy1}=-\frac{V_{cy}-V_{rx}}{V_{Lxy}}R_{L}=-\frac{V_{in}+(\frac{V_{CG}}{R_{CG}}+\frac{V_{Lxy}}{R_{L}})R_{sc}+\frac{V_{Lxy}}{R_{L}}R_{sr}}{V_{Lxy}}R_{L}$$

where *V_CG_* is the output voltage of the *Amp2*.

In Equation (6), *V_in_*, *R_CG_*, and *R_L_* are known, *R_sc_* and *R_sr_* of the multiplexers can be known from the datasheet, and *V_CG_* and *V_Lxy_* can be measured with the analog-to-digital converter (ADC). Therefore, the voltage drops on the switch-on resistances of the scanning row multiplexer and the scanning column multiplexer are eliminated, and then the accurate voltage drop on the EBT can be obtained. Thus the precise resistance of the EBT in the improved circuit can be calculated with Equation (6).

## 3. Experiments and Discussion

### 3.1. Simulation Experiments

We investigated the effects of the *R_switch_*, the non-scanned element (*R_non-scanned_*), the row number (*M*), and the column number (*N*) on the basic circuit and the proposed improved circuit in the 2-D resistive sensor array using National Instrument Multisim 12 (National Instrument Corporation, Austin, TX, USA). In the simulation experiments, a precise op-amp, OPA2340 (Burr-Brown Corporation, Tucson, AZ, USA), was selected as the macro-model of the op-amp. In the simulation experiments, the *V_in_* was −5.0 V, *R_CG_* and *R_L_* were 50 Ω and 1 kΩ, respectively.

#### 3.1.1. Effect of the Multiplexers’ Switch-on Resistance

**Figure 2:**
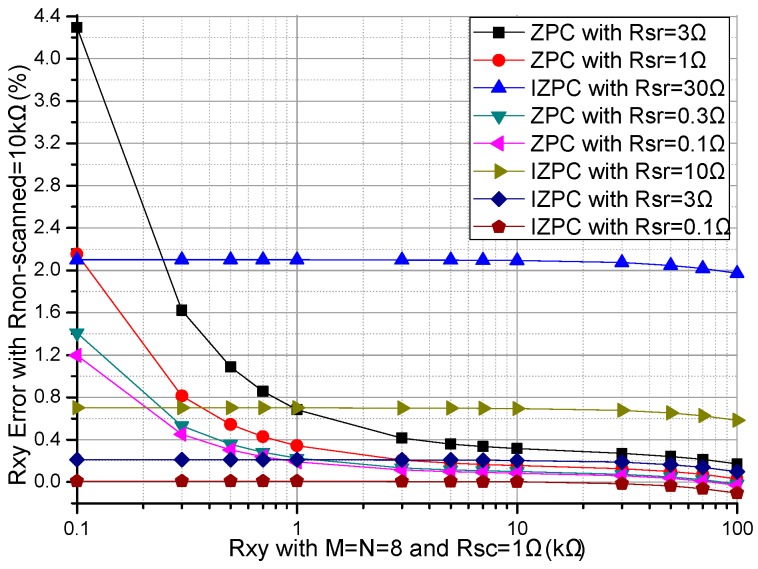
Effect of *R_sr_* on the *R_xy_* errors of the basic circuit and those of the improved circuit.

**Figure 3:**
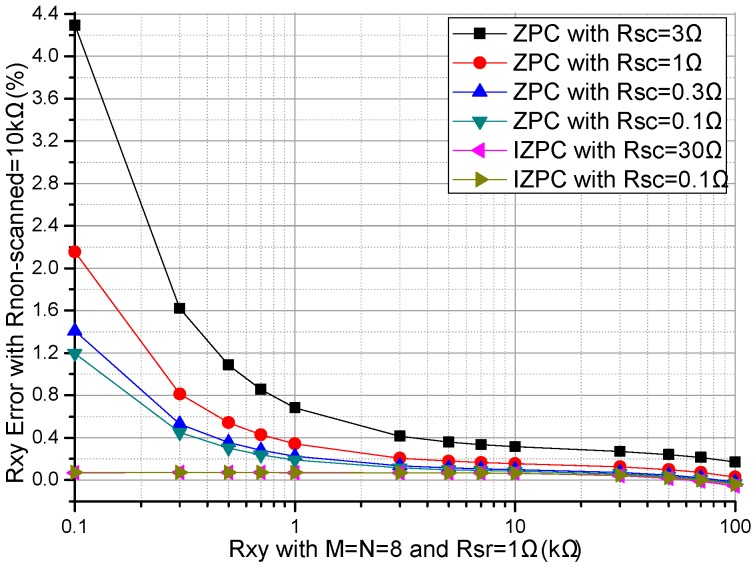
Effect of *R_sc_* on the *R_xy_* errors of the basic circuit and those of the improved circuit.

The performances of the ZPCs in the 2-D resistive sensor arrays were affected by the multiplexers’ switch-on resistances including the row multiplexer’s switch-on resistance (*R_sr_*) and the column multiplexer’s switch-on resistance (*R_sc_*) [17,26]. The multiplexers had the switch-on resistances of several hundred milliohms to several hundred ohms [25], which would have less effect on the crosstalk of the resistive sensor arrays with smaller switch-on resistance. In simulations, we fixed some parameters, including the resistances of all *R_non-scanned_*s in the resistive sensor array at 10 kΩ, and *M* and *N* at 8. In the *R_sr_* experiment, all *R_sc_*s were fixed at 1 Ω, all *R_sr_*s in the basic circuit varied synchronously with the same resistance value from 0.1–3 Ω and all *R_sr_*s in the improved circuit varied synchronously with the same resistance value from 0.1–30 Ω, the resistance value of the EBT varied in the range from 0.1–100 kΩ and the experiment results of two ZPCs were shown in Figure 2. In the *R_sc_* experiment, all *R_sr_*s were fixed at 1 Ω, all *R_sc_*s in the basic circuit varied synchronously with the same resistance value from 0.1–3 Ω and all *R_sc_*s in the improved circuit varied synchronously with the same resistance value from 0.1–30 Ω, the resistance value of the EBT varied in the range from 0.1–100 kΩ and the experiment results of two ZPCs were shown in Figure 3.

From Figure 2, with *R_sr_* varied from 0.1–3 Ω, the *R_xy_* errors in the basic circuit showed a large change (from 1.20% to 4.29% at *R_xy_* of 0.1 kΩ) which was more obvious with the smaller *R_xy_*; with *R_sr_* varied from 0.1–30 Ω, the *R_xy_* errors in the improved circuit showed small variations (from 0.01% to 2.10% at *R_xy_* of 0.1 kΩ); with the increase of *R_xy_*, the *R_xy_* errors in the basic circuit showed an obvious negative coefficient, which was more obvious with *R_xy_* of the smaller resistance; the *R_xy_* errors in the improved circuit showed a tiny negative coefficient, which was a little obvious with *R_xy_* of the larger resistance.

From Figure 3, with *R_sc_* varied from 0.1–3 Ω, the *R_xy_* errors in the basic circuit showed a large change (from 1.20% to 4.29% at *R_xy_* of 0.1 kΩ) which was more obvious with the smaller *R_xy_*; with *R_sc_* varied from 0.1–30 Ω, the *R_xy_* errors in the improved circuit showed tiny variations (from −0.06% at *R_xy_* of 100 kΩ to 0.07% at *R_xy_* of 0.1 kΩ); with the increase of *R_xy_*, the *R_xy_* errors in the basic circuit showed an obvious negative coefficient, which was more obvious with *R_xy_* of a smaller resistance; with the increase of *R_xy_*, the *R_xy_* errors in the improved circuit showed a negligible change.

From Figure 2 and Figure 3, *R_sr_* and *R_sc_* had similarly large effects on the *R_xy_* errors of the basic circuit, while *R_sr_* had a small effect and *R_sc_* had a tiny effect on the *R_xy_* errors in the improved circuit.

#### 3.1.2. Array Size Effect Experiment

**Figure 4:**
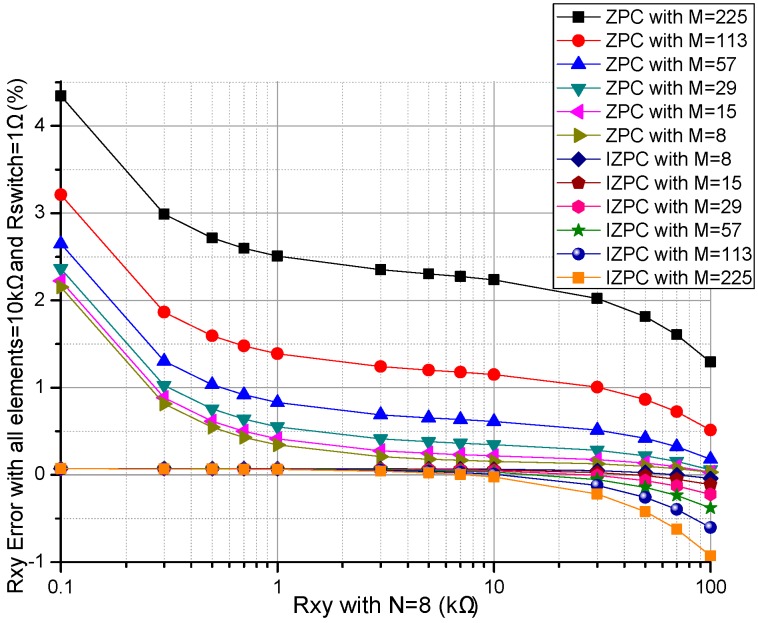
Effect of *M* on the *R_xy_* errors of the basic circuit and those of the improved circuit.

**Figure 5:**
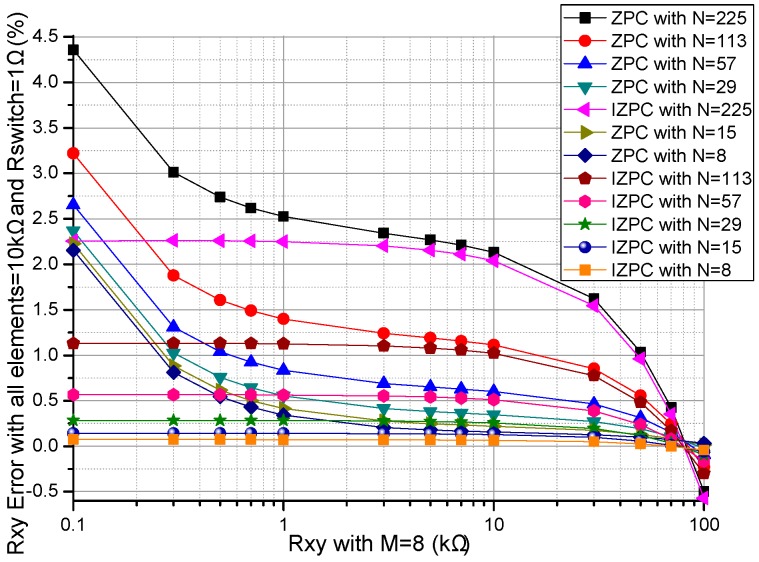
Effect of *N* on the *R_xy_* errors of the basic circuit and those of the improved circuit.

Array size, such as the row number (*M*) and the column number (*N*), were proved to affect the performance of the ZPCs in the *M* × *N* resistive sensor arrays [17,26]. We investigated the effect of *M* and *N* on two ZPCs. In simulations, we fixed some parameters including the resistances of all *R_non-scanned_*s in the resistive sensor array at 10 kΩ, all *R_switch_*s at 1 Ω, *M* or *N* at 8, *N* or *M* was one number selected from 8, 15, 29, 57, 83, 98, 113, and 225. The array size effects on the basic circuit and the proposed improved circuit were simulated in NI Multisim and the results were shown in Figure 4 and Figure 5.

From Figure 4, with the increase of *M*, the *R_xy_* errors in the basic circuit showed a positive coefficient while the *R_xy_* errors in the improved circuit showed a negative coefficient, which was more obvious with the larger *M* and the larger *R_xy_*. With the increase of *R_xy_*, the *R_xy_* errors in the basic circuit showed an obvious negative coefficient, and the *R_xy_* errors in the improved circuit also showed a small negative coefficient, which was more obvious with the larger *R_xy_*.

From Figure 5, with the increase of *N*, both the *R_xy_* errors in the basic circuit and the *R_xy_* errors in the improved circuit showed positive coefficients, in which the *R_xy_* errors in the improved circuit were less than the *R_xy_* errors in the basic circuit for the same *R_xy_*. With the increase of *R_xy_*, the *R_xy_* errors in the basic circuit showed an obvious negative coefficient, and the *R_xy_* errors in the improved circuit showed a small negative coefficient, which was more obvious with the larger *R_xy_*.

From Figure 4 and Figure 5, *M* and *N* had similar effects on the *R_xy_* errors in the basic circuit, while *M* and *N* have different effects on the *R_xy_* errors in the improved circuit. In the improved circuit, *N* had more obvious effect on the *R_xy_* errors than *M* did.

#### 3.1.3. Effect of the Resistances of the Non-Scanned Elements

**Figure 6:**
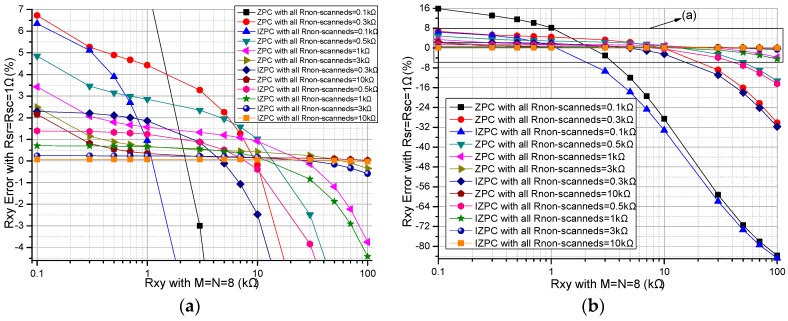
Effect of *R_non-scanned_* on the *R_xy_* errors of the basic circuit and those of the improved circuit: (**a**) the partial enlarged view; and (**b**) full view.

All non-scanned elements (*R_non-scanned_*s) affected the ZPCs’ performance in the resistive sensor arrays [17,26]. We investigated the *R_non-scanned_*’s effect on two ZPCs. In simulations, we fixed some parameters including all *R_switch_*s at 1 Ω, and *M* and *N* at 8. All *R_non-scanned_*s of two ZPCs had the same resistance of 0.1 kΩ, 0.3 kΩ, 0.5 kΩ, 1 kΩ, 3 kΩ, or 10 kΩ, the results are shown in Figure 6, and Figure 6a is an enlarged view of the part in Figure 6b.

From Figure 6, with the same resistance of all *R_non-scanned_*s, the improved circuit had a wider range than the basic circuit did. The *R_non-scanned_*’s effect on the *R_xy_* errors in the basic circuit was more obvious than its effect on the *R_xy_* errors in the improved circuit. With the increase of *R_xy_*, both the *R_xy_* errors in the basic circuit and those in the improved circuit showed obvious negative coefficients, which were more obvious for the larger *R_xy_*.

#### 3.1.4. Effects of Array Size, the Non-Scanned Element, and the Multiplexer’ Switch-On Resistance on the Currents

**Figure 7:**
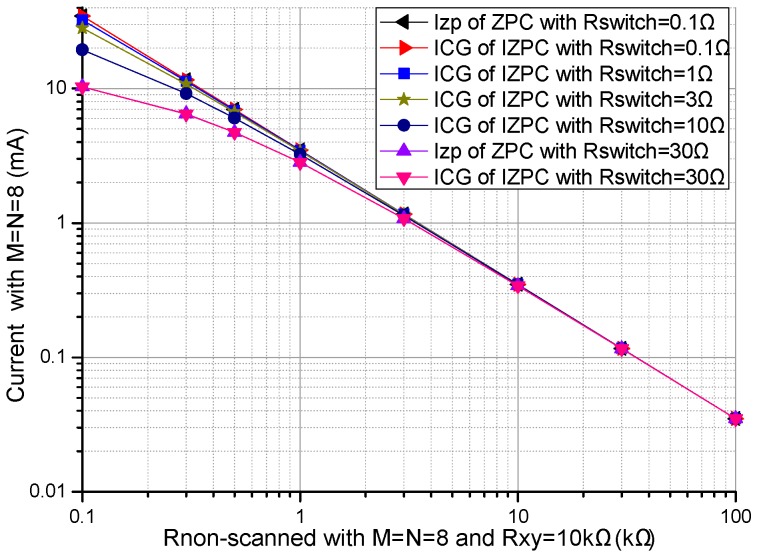
Effect of *R_non-scanned_* and *R_switch_* on the currents of the basic circuit and those of the improved circuit.

**Figure 8:**
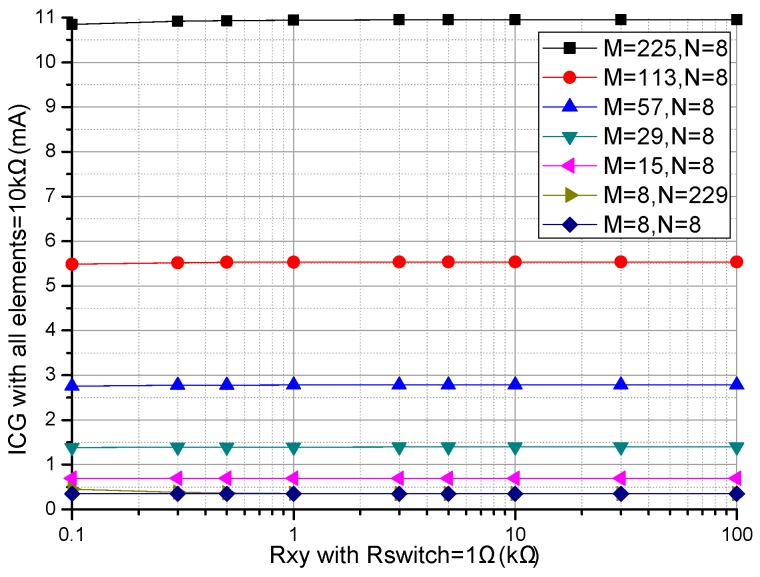
Effect of array size on the *I_CG_* of the improved circuit.

We investigated the effect of non-scanned element (*R_non-scanned_*) and the multiplexers’ switch-on resistance (*R_switch_*) on the total bypass current (*I_zp_*) in the basic circuit and the effect of array size, *R_non-scanned_* and *R_switch_* on the *R_CG_*’s current (*I_CG_*) in the improved circuit. In simulations, we fixed some parameters including *R_xy_* at 10 kΩ, and *M* and *N* at 8. In *R_non-scanned_* and *R_switch_* effect experiments, results of the *I_CG_* and the *I_zp_* are shown in Figure 7. In array size effect experiments, results of the *I_zp_* are shown in Figure 8.

From Figure 7, with the decrease of all *R_non-scanned_*s and the decrease of the all *R_switch_*s, both the *I_CG_* and the *I_zp_* increased, which were more obvious for *R_non-scanned_* with the smaller resistance; with the variations of *R_non-scanned_* and *R_xy_*, the difference between the *I_CG_* and the *I_zp_* was very tiny. Therefore, the *I_CG_* in the improved circuit had similar feature as the *I_zp_* in the basic circuit with the variations of *R_non-scanned_* and *R_xy_*.

From Figure 8, *N* had a tiny effect, while *M* had an obvious effect on the *I_CG_* in the improved circuit. With the increase of *M*, there was a linear increase of the *I_CG_* in the improved circuit.

### 3.2. Test Experiments with the Prototype Circuit

**Figure 9:**
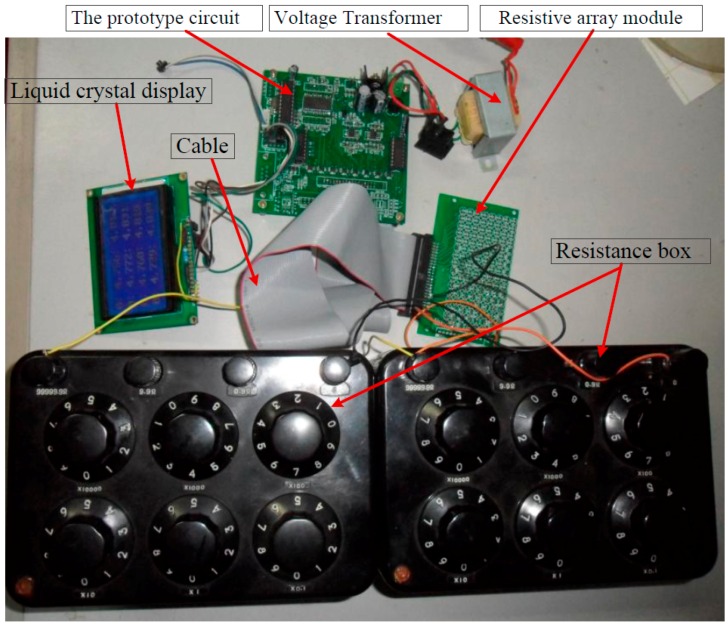
The experimental setup of the prototype circuit.

**Figure 10:**
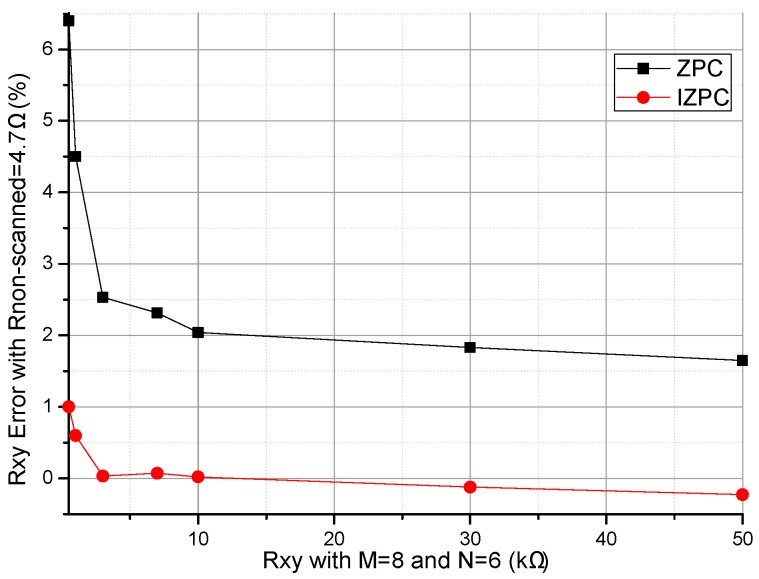
Result of EBT varied within 500–50 kΩ in the prototype circuit.

**Figure 11:**
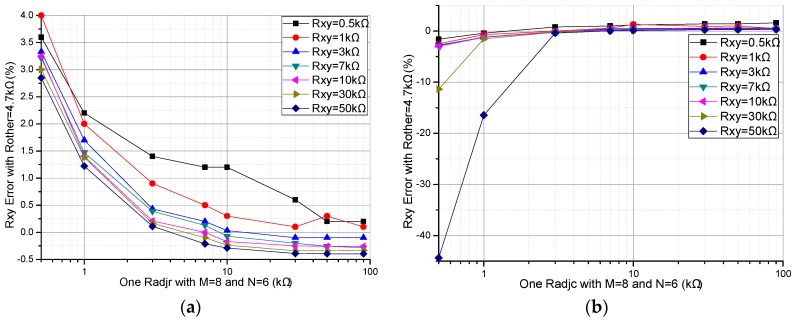
Results of one adjacent element on the EBT’s errors of the improved circuit: (**a**) one *R_adjr_*; and (**b**) one *R_adjc_*.

A prototype circuit was designed and the experimental setup is shown in Figure 9. In the prototype circuit, the EBT’s resistance (*R_xy_*) of the basic circuit was calculated with Equation (2) and the EBT’s resistance (*R_xy_*_1_) of the improved circuit was calculated with Equation (6). In the prototype circuit, OPA2376 (Texas Instruments Incorporated, Dallas, TX, USA) (from the datasheet, the offset voltage, the bias current, the gain-bandwidth, and the gain are equal to 5 μV, 0.2 pA, 5.5 MHz, and 134 dB, respectively) was used as the op-amp, ADG884 (Analog Devices Inc., Norwood, MA, USA) (from the datasheet, the maximum on-resistance, the maximum on-resistance match between channels, and the maximum on-resistance flatness are equal to 0.41 Ω, 0.05 Ω, and 0.15 Ω, respectively) was used as the multiplex switch, and *R_CG_* and *R_L_* were 100 Ω and 1 kΩ, respectively. In the prototype circuit, *M* and *N* were 8 and 6, respectively. A cable with the length of 400 mm and 14 core lines was used to connect the resistive sensor array modules with the circuit. For avoiding bipolar power, the *V_zp_* was connected to a constant offset voltage (0.500 V) and the *V_in_* was connected to ground. Thus, the equivalent *V_in_* in the prototype circuit was −0.500 V. In the test experiments, every varied element was replaced by the precision resistance box with its smallest step resistance value at 0.1 Ω, and all other elements were resistors at 4.7 kΩ. With all non-scanned elements (*R_non-scanned_*) fixed at 4.7 kΩ and the EBT varied from 0.5 kΩ to 50 kΩ, the results in the basic circuit and those of the improved circuit were shown in Figure 10. With EBT fixed at 0.5 kΩ, 1 kΩ, 3 kΩ, 7 kΩ, 10 kΩ, 30 kΩ, 50 kΩ, or 90 kΩ, the EBT errors in the improved circuit for one row adjacent element (*R_adjr_*) varied from 0.5 kΩ to 50 kΩ were shown in Figure 11a and the EBT errors for one column adjacent element (*R_adjc_*) varied from 0.5 kΩ to 50 kΩ were shown in Figure 11b.

From the results of the prototype circuit in Figure 10, we found that the EBT’s error of the improved circuit was less than that of the basic circuit in a wide resistance range. Additionally, we found that the measurement result was unstable when the EBT had a larger resistance value (*R_xy_* > 50 kΩ). The reason could be the nonlinear output of the prototype circuit and the limited resolution of the ADC used in it.

From the results in Figure 11, we found that the EBT errors decreased with the increase of one *R_adjr_* and the EBT errors increased with the increase of one *R_adjc_*. From the results in those in Figure 11b, we found that the *R_adjc_* with small resistance (<3 kΩ) had a significant effect on the EBT errors when the EBT’s resistance was large (>30 kΩ).

## 4. Discussion

As shown in Figure 1a, the basic ZPC [17] with the simplest structure had one voltage feedback op-amp and one feedback resistor, but it suffered from *R_switch_*s’ crosstalk. The crosstalk caused by *R_switch_*s was partly suppressed by the IIDFC [15] with a simpler structure and the crosstalk caused by *R_switch_*s was completely suppressed by the IIDFC with compensation [16], in which two sampling channels were used. The crosstalk for long cables and *R_switch_*s was well suppressed by the two-wire circuits, including the two-wire VFC [22], the two-wire S-NSDE-EPC [23], the multi-channel part two-wire ZPC [24], and the multi-channel full two-wire ZPC [25]. However, in these two-wire circuits, more extra components and more sampling channels were necessary. Features of ZPCs and those of VFCs were listed in Table 1. As shown in Figure 1b, the improved ZPC had two voltage feedback op-amps and two feedback resistors. Thus, one more op-amp, one more resistor, and one more sampling channel were used in the proposed circuit. At the same time, more calculations were necessary in it.

With the switch-on resistances of several hundred milliohms to several hundred ohms [25], *M* + *N* 2:1 multiplexers were necessary for the basic circuit and the improved circuit in the *M* × *N* resistive sensor array. From Figure 2 and Figure 3, the EBT’s errors caused by the multiplexers’ switch-on resistances in the basic circuit were larger than those in the improved circuit. Therefore, in the improved circuit, the EBT’s errors caused by the *R_sr_* and the *R_sc_* were reduced greatly, in which the *R_sc_* had a smaller effect. Thus the 2:1 multiplexers with larger switch-on resistances could be used in the improved circuit with good performance.

With the increase of the array size, including the row number (*M*) and the column number (*N*), the EBT’s bypass current in the parallel path would increase, which caused obvious crosstalk in the basic circuit [17,26]. From Figure 4 and Figure 5, the EBT’s errors caused by *M* and *N* were reduced greatly in the improved circuit, in which *M* had less effect on the EBT’s errors. Thus the larger array size can be used in the resistive sensor array with the improved circuit.

From Figure 6, all non-scanned elements as parallel paths affected the performances of the basic circuit and the improved circuit. With the increase of *R_non-scanned_*, the change of the *R_xy_* errors in the improved circuit was less than the change of the *R_xy_* errors in the basic circuit. With all non-scanned elements at the same resistance of the smaller value, the improved circuit has better measurement accuracy than the basic circuit. Thus, the improved circuit can be used in the resistive sensor array with each element of smaller resistance.

From Figure 7, with the same *R_non-scanned_* and the same *R_xy_*, the difference between the *I_CG_* in the improved circuit and the *I_zp_* in the basic circuit was very tiny. With the decrease of all *R_non-scanned_*s and the decrease of the all *R_switch_*s, both the *I_CG_* and the *I_zp_* increased. From Figure 8, there was a linear increase of the *I_CG_* with the increase of *M*, but there was a tiny increase of the *I_CG_* with the increase of *N*. Therefore, in the array with sensitive elements of smaller resistances and the larger row number, larger currents and op-amps with larger current driving ability are necessary for both the basic circuit and the improved circuit, in which larger currents can cause greater power consumption. Thus, the larger column number is better for low power consumption and small current driving requirement of the op-amps in the resistive sensor array with the improved circuit. As the *I_CG_* and the *I_L_* are all currents consumed in the resistive sensor array, power consumption in the resistive sensor array with the improved circuit can be calculated with the *I_CG_* and the *I_L_*.

From Figure 10, the improved circuit showed better performance than the basic circuit did. From Figure 11, the *R_adjr_* and the *R_adjc_* showed opposing effects in the prototype circuit. However, the real test result is not as good as the simulated result, which may be caused by the non-ideal performances of the op-amp and the multiplexer in the prototype circuit.

The resistance value of the force sensing resistor (ThruMode Matrix Array of Sensitronics, Bow, WA, USA) was in the range of 1–20 kΩ for pressure in 1–16 PSI [27] and the resistance value of the force-sensing resistor (ShuntMode Matrix Array of Sensitronics) was in the range of 7–100 kΩ for pressure in 7–85 PSI [28]. Thus resistance values in the array were set in the range of 0.1–100 kΩ in the simulated experiments. There were a number of sensitive elements in the arrays with their resistance lower than 0.1 kΩ and higher than 100 kΩ. At the same time, the resistance values in the chemical sensor arrays could be greatly different, for example a range of six decades [29]. In the ZPC, the array’s element with low resistance could cause a high current, which was still a challenge in most readout circuits for their op-amp’s limited current driving capability. Thus, a new readout circuit with wide resistance range should be developed for the resistive sensor arrays used in tactile sensor and chemical sensor.

In some ZPCs [17,20,21,22,23] including the S-NSE-ZPC, the S-NSSE-ZP circuit, and the S-NSDE-ZP circuit, each with one op-amp in negative feedback, could access only one element in the 2-D resistive sensor arrays at the same time. In this paper, the improved S-NSE-ZPC was given as an example for verifying the performance of the improved zero potential circuit, in which the current from zero potential to the array was sampled and used to calculate the EBT’s precision resistance. A similarly improved method may also be suitable for the S-NSSE-ZPC and the S-NSDE-ZPC. As for the fast readout rate ZPCs [11,19] with many sampling op-amps in negative feedback, the improved method would also be useful, but their performance should be verified with experiments.

## 5. Conclusions

Firstly, the improved zero potential circuit of the 2-D resistive sensor array was proposed in this paper. Then, by analytical conduction, the improved S-NSE-ZPC was given as an example for verifying the performance of the improved ZPC. A similarly improved method may also be suitable for the S-NSSE-ZP circuit and the S-NSDE-ZP circuit. Then the effects of the multiplexer’s switch-on resistance, the column number, the row number, and the non-scanned elements’ resistances on the measurement accuracy of the elements being tested in the basic circuit and those of the improved circuit were verified with experiments. The experimental results show that, in the 2-D resistive sensor array with the improved zero potential circuit, the effects of the switch-on resistances, the row number, the column number, and the non-scanned elements’ resistances on the measurement error of the element being tested have been reduced greatly; a larger row number is preferred for good accuracy, and a larger column number is better for low power consumption and small current driving requirements of the op-amp in the 2-D resistive sensor array with the improved ZPC.

## Figures and Tables

**Table 1:** Comparison of ZPCs and VFCs of the *M* × *N* resistive sensor arrays.

Methods	Auxiliary Components	Advantages	Disadvantages
Basic ZPC [17]	*M* + *N* multiplexers, one op-amp, one resistor, and one sampling channel	Simplest structure	*R_switch_*s’ crosstalk, Low readout rate
IIDFC [15]	*M* + *N* multiplexers, one op-amp, three resistors, and one sampling channel	Simpler structure, *R_switch_*s’ crosstalk partly suppressed	Part *R_switch_*s’ crosstalk, Low readout rate
IIDFC with Compensation [16]	*M* + *N* multiplexers, one op-amp, four resistors, and two sampling channels	*R_switch_*s’ crosstalk suppressed, and simple structure,	Low readout rate
Two-wire VFC [22]	*2M* + *N* multiplexers, *N* + 1 op-amps, one resistor, and two sampling channels	Cable’s crosstalk suppressed, *R_switch_*s’ crosstalk suppressed,	Complex structure, Low readout rate
Two-wire ZPC [23]	*2M* + *N* multiplexers, *M* + *N* op-amps, three resistors, and two sampling channels	Cable’s crosstalk suppressed, *R_switch_*s’ crosstalk suppressed,	Complex structure, Low readout rate
Multi-channel part Two-wire ZPC [24]	*M* multiplexers, *N* + 1 op-amps, *N* resistors, and *N* sampling channels	Better accuracy, and fastest readout rate	More complex structure
Multi-channel full 2-wire ZPC [25]	*N* multiplexers, M op-amps, *M* resistors, and *M* sampling channels	Best accuracy, and fastest readout rate	Most complex structure
Proposed	*M* + *N* multiplexers, two resistors, and two sampling channels	*R_switch_*s’ crosstalk suppressed, simple structure, and estimation of array’s power	Low readout rate
